# Anticancer Effect of Mountain Ginseng on Human Breast Cancer: Comparison with Farm-Cultivated Ginseng

**DOI:** 10.1155/2020/2584783

**Published:** 2020-07-25

**Authors:** Jungeun Kim, Jae-Myung Yoo, Jin Soo Kim, Sun-Gun Kim, Jong Eel Park, Young Mi Seok, Jun-Ho Son, Hyo Jung Kim

**Affiliations:** ^1^Korean Medicine R&D Team 1, National Institute for Korean Medicine Development, Gyeongsan 38540, Republic of Korea; ^2^Unimed Pharmaceutical Inc., Seoul 05567, Republic of Korea

## Abstract

Mountain ginseng has been used generally as a pharmacopuncture for cancer therapy in clinical practice in Northeast Asia. Nonetheless, there have been few scientific reports for the anticancer action of mountain ginseng. In this study, we investigated whether mountain ginseng extract (MGE) could inhibit the growth of breast cancer in *in vitro* and *in vivo* models. MGE showed stronger cytotoxicity than farm-cultivated ginseng extract (FGE) through promoting ROS generation. Also MGE dose-dependently brought about mitochondrial dysfunction in MCF-7 cells. In addition, MGE induced apoptosis through enhancing the activities of caspase-3/7 by regulation of expression of Bcl-2, Bax, cytochrome c, and cleaved caspase-3 in the MCF-7 cells. Consistent with the *in vitro* results, MGE significantly reduced tumor weights compared with FGE in mice transplanted with MCF-7 cells, and it regulated the expression of apoptosis-related proteins, such as Bcl-2, Bax, cytochrome c, cleaved caspase-3, and cleaved PARP, in the tumor tissues. Additionally, MGE included higher total ginsenoside contents than FGE. In conclusion, MGE, which is richer in ginsenosides, exerts a stronger anticancer action than FGE in breast cancer. The anticancer action of MGE may be closely correlated with caspase-mediated apoptosis through upregulating ROS generation. Therefore, these findings may be helpful for a clinical understanding of the anticancer mechanism of MGE for breast cancer patients.

## 1. Introduction

Ginseng, affiliated to the Araliaceae family, is a perennial plant, and it is mainly distributed in Korea, China, Japan, and North America [1]. Among various ginseng species, especially *Panax ginseng* has been called Korean ginseng. Korean ginseng has been widely used as a component of traditional medicine and in functional foods worldwide because it exerts many beneficial effects, such as antioxidant, anticancer, antidiabetes, anti-inflammation, and neuroprotection [2–4]. The beneficial effects of ginseng are closely associated with its bioactive components including ginsenosides, phenolic acids, flavonoids, and polysaccharides [2, 4]. In particular, Korean ginseng, which grows in the mountains, is called mountain ginseng in Northeast Asia, and it is known that mountain ginseng includes higher amounts of bioactive compounds than farm-cultivated ginseng [5]. However, the effect of mountain ginseng on biological events is almost unknown.

Breast cancer is one of the most common cancers that develop in women, and it has the highest incidence and mortality rates in the world [6]. Most breast cancers are known to belong to the estrogen-dependent type [6]. Recently, in Korea, the five-year survival rate of breast cancer patients has been the highest among those of other cancers, except for thyroid cancer [7]. This is because therapy methods, such as surgery, radiotherapy, and chemotherapy, for breast cancer patients have been developed rapidly, while breast cancer has been mainly found in the early stages by the government-organized National Health Insurance Service [8]. However, the mortality rate of breast cancer patients has been steadily increasing since a statistical survey of cancer generation was conducted in 1999 [7] because the five-year survival rates of breast cancer patients (stages 3 and 4) are still low [9]. The patients mainly receive radiotherapy and chemotherapy treatments because metastatic cancer (state 4) is never completely removed by surgical treatment. As a result, many patients suffer pain from both the side effects of radiotherapy and chemotherapy and cancer recurrence with anticancer drug resistance. In Korea, some patients, including terminally ill patients, have received pharmacopuncture treatment with mountain ginseng extract (MGE) in combination with or without anticancer drugs for reduction of pain and side effects [10]. Nonetheless, preclinical scientific evidence related to the anticancer effect of MGE on breast cancer has not been reported.

In this study, we investigated whether MGE could inhibit the growth of human breast cancer in *in vitro* and *in vivo* models, and we found that MGE showed stronger anticancer action than farm-cultivated ginseng extract (FGE). In addition, we revealed the molecular mechanism of the anticancer action of MGE in human breast cancer. The findings may provide novel information for the clinical application of MGE as an anticancer herbal drug for breast cancer therapy, and they may be helpful for understanding the anticancer mechanism of MGE.

## 2. Materials and Methods

### 2.1. Materials

DMEM, 1 × PBS, antibiotics, and FBS were obtained from GE Healthcare Life Sciences (Hyclone, Logan, UT, USA). Specific antibodies against Bcl-2 (B-cell lymphoma 2, #15071), Bax (Bcl-2-associated *X* protein, #2774), cytochrome c (#4272), cleaved caspase-3 (#9661), PARP (poly (ADP-ribose) polymerase, #9542), and cleaved PARP (#5625) were purchased from Cell Signaling Technology, Inc. (Beverly, MA, USA). A specific antibody against *β*-actin (sc-1616) was procured from Santa Cruz Biotechnology, Inc. (Dallas, TX, USA). ELISA kit for caspase-3/7 activity was obtained from Promega, Corp. (Madison, WI, USA). Ginsenoside standards were procured from ChemFaces Co., Ltd. (Wuhan, China). Dihydroethidium (DHE) was obtained from Cayman Chemical Company, Inc. (Ann Arbor, MI, USA). 2′,7′-Dichlorodihydrofluorescein diacetate (DCFDA), 3-(4,5-dimethylthiazol-2-yl)-2,5-diphenyltetrazolium bromide (MTT), tetraethylbenzimidazolylcarbocyanine iodide (JC-1), and all other chemicals were purchased from Sigma-Aldrich (St Louis, MO, USA). All other chemicals were of analytical grade.

### 2.2. Preparation of Mountain Ginseng and Farm-Cultivated Ginseng Extracts

Mountain ginseng, certified by the president of the Korea Forestry Promotion Institute, and farm-cultivated ginseng were purchased from a farmhouse (Chuncheon, Korea) and Omniherb Co., Ltd. (Daegu, Korea), respectively. The herbs were identified by Dr. H. Lee, a herbalist, and deposited in the National Institute for Korean Medicine Development (NIKOM, Gyeongsan, Korea). To prepare MGE and FGE, dried fragments of their roots (100 g) were boiled in 30% ethanol solution (1 liter) for approximately 3 h and then deposited overnight at 4°C after cooling. The collected supernatant was filtered through a 0.45 *μ*m filter, and the filtrate was concentrated by an evaporator (N-1300, Sunileyela Co., Ltd, Korea) and then lyophilized. The dried pellets were stored at −20°C until use. MGE and FGE were dissolved in 4% ethanol solution for *in vitro* and *in vivo* experiments.

### 2.3. High-Performance Liquid Chromatography Analysis

Ginsenoside contents in MGE and FGE were analyzed using a HPLC system (e2695 Separations Module) equipped with a HPLC pump, an autosampler, a column oven, and a diode array UV/Vis detector (2998 PDA detector; Waters Corp., Milford, MA, USA) with a C_18_ column (4.6 × 250 mm, 5 *μ*m; YMC Co., Ltd., Kyoto, Japan). The ginsenosides were eluted in a gradient system composed of solvent A (0.2% phosphoric acid) and solvent B (acetonitrile). The gradient was 80-80-77-70-60-50-15-15-80-80% of solvent A at the gradient time *t* G = 0-5-20-25-30-35-60-62-65-70 min, respectively. The temperature of the column oven was 30°C, and the flow rate was 1.0 ml/min; an injection volume of 10 *μ*l was applied. The UV/Vis detector was set at the wavelength range of 203 nm. The ginsenoside standards and the sample solution (0.1 mg/ml) were dissolved and diluted in methanol. The relative standard deviation of the measured concentrations was used to assess precision. A comparison of the mean measured concentration versus the corresponding nominal concentration was used to assess accuracy.

### 2.4. Animals

BALB/*c* athymic nude mice (female, 6 weeks, and 19–21 g) were obtained from Koatech (Pyeongtaek, Korea) and housed in cages (5 mice per cage) under specific pathogen-free conditions (21°C–24°C and 40–60% relative humidity) with a 12 h light/dark cycle. They were given free access to standard rodent food (Envigo, Madison, WI, USA) and water. All animal experiments were approved by the Committee of Animal Care and Experiment of NIKOM with a reference number (NIKOM-2019-003). Animal studies were performed according to the guidelines of the Animal Care and Use Committee at NIKOM.

### 2.5. Xenograft Model

Xenograft model was carried out according to a modification of a protocol previously reported [11]. After adaptation, MCF-7 cells, a breast cancer cell line originating from human breast adenocarcinoma [12], in Matrigel mixture (3 × 10^6^ cells/mouse) were injected into mammary glands of mice. During the experimental period, the mice were intravenously given MGE (50 or 100 mg/kg) or FGE (100 mg/kg) once a day for 4 weeks, and their body weights and food intake were monitored once a week. On the final day, tumor tissues were isolated from sacrificed mice and then washed with cold 1 × PBS twice after measurement of tissue weights. The washed tumor tissues were used for histological analysis and immunoblot analysis.

### 2.6. Histological Analysis

Histological analysis was performed by following a modification of a previous method [13]. Briefly, deparaffinized tumor tissues on slices were stained with hematoxylin-eosin or incubated overnight at 4°C with a 1 : 100 dilution of specific antibodies against Bax or cleaved PARP. Next day, the tissue slices were incubated with anti-mouse/rabbit antibodies conjugated with horseradish peroxidase for 2 h, incubated with diaminobenzidine solution, and then counterstained with hematoxylin. Finally, all the stained tissue slices were embedded with mounting solution. Histological changes of tumor tissues were observed under a light microscope with 200x magnification.

### 2.7. Cell Culture

MCF-7 cells were obtained from Korean Cell Line Bank (Seoul, Korea). The cells were cultured in DMEM medium containing 10% (v/v) FBS and antibiotics at 37°C in a humidified atmosphere of 5% CO_2_. All *in vitro* tests contain a vehicle control group (0.016% ethanol).

### 2.8. Cell Viability Assay

Cell viability was determined following a modification of a method reported previously [14]. Briefly, MCF-7 cells were seeded on a 96-well plate (5 × 10^3^ cells/well) in DMEM with 10% FBS and then incubated for 24 h. The cells were incubated with MGE or FGE (0–400 *μ*g/ml) for 22 h and then further incubated with 200 *μ*l culture media containing 500 *μ*g/ml MTT reagent for 2 h. Next, 100 *μ*l of dimethyl sulfoxide was added to the plate after supernatant was removed and then incubated for 15 min. Cell viability was determined at 570 nm using a microplate reader (Sunrise, TECAN, Mӓnnedorf, Switzerland).

### 2.9. Determination of Intracellular Reactive Oxygen Species and Mitochondrial Dysfunction

After treatment of MGE or FGE, MCF-7 cells were stained with 10 *μ*M DHE, DCFDA, or JC-1 in Hank's balanced salt solution for 30 min in the darkness. The cells were observed under a fluorescence microscope with 200x magnification. The density of each image was measured using ImageJ Software (Version 1.51j8 for Windows; NIH, USA).

### 2.10. Enzyme-Linked Immunosorbent Assay of Caspase-3/7 Activity

To measure the caspase-3/7 activity in cell lysate, cell lysates were centrifuged and then stored at −80°C until use. The caspase-3/7 activity was detected by using an ELISA kit according to the manufacturer's instructions.

### 2.11. Immunoblot Analysis

Immunoblot analysis was evaluated following a method reported previously [11]. Briefly, the blotted proteins on PVDF membrane were visualized using a chemiluminescent reaction (Immobilon Western, Millipore Corp., Billerica, MA, USA) with an Imaging system (ImageQuant LAS 4000, GE Healthcare Life Sciences, Marlborough, MA, USA). The level of target proteins was compared with that of a loading control (*β*-actin), and the results were expressed as a ratio of density of each protein identified by a protein standard size marker (BIOFACT Co., Ltd., Daejeon, Korea). The density of each inverted band was measured using ImageJ software.

### 2.12. Statistical Analyses

The experimental results were listed as means ± SD for *in vitro* studies or SEM for *in vivo* studies. One-way and two-way analysis of variance (ANOVA) was used for multiple comparisons (GraphPad Prism version 5.03 for Windows, San Diego, CA, USA). We applied the Dunnett or Tukey's test for one-way ANOVA and the Bonferroni posttest for two-way ANOVA for significant variations between treated groups. Differences at ^*∗*^*P* < 0.05 and ^*∗∗*^*P* < 0.01 levels were considered statistically significant.

## 3. Results

### 3.1. Effects of MGE and FGE on Cell Viability, ROS Generation, and Mitochondrial Dysfunction in MCF-7 Cells: Comparison between MGE and FGE

**Figure 1:**
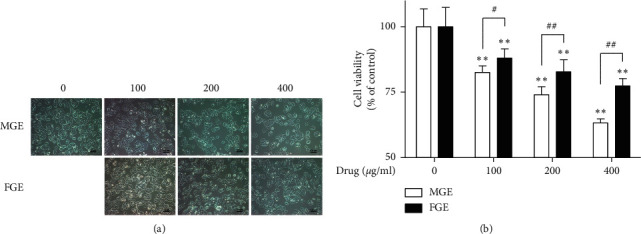
Inhibitory effects of MGE and FGE on cell viability of MCF-7 cells. MCF-7 cells were seeded on a 96-well plate (5 × 10^3^ cells/well) in DMEM with 10% FBS and then incubated for 24 h. The cells were incubated with MGE or FGE (0–400 *μ*g/ml) for 22 h. Cell viability was determined as described in the Materials and Methods section. Data are expressed as the mean ± SD values of sextuple determinations. ^*∗*^*P* < 0.05 and ^*∗∗*^*P* < 0.01 versus the control group. ^#^*P* < 0.05 and ^##^*P* < 0.01 versus the same concentration group. (a) Cell morphology; (b) cell viability.

**Figure 2:**
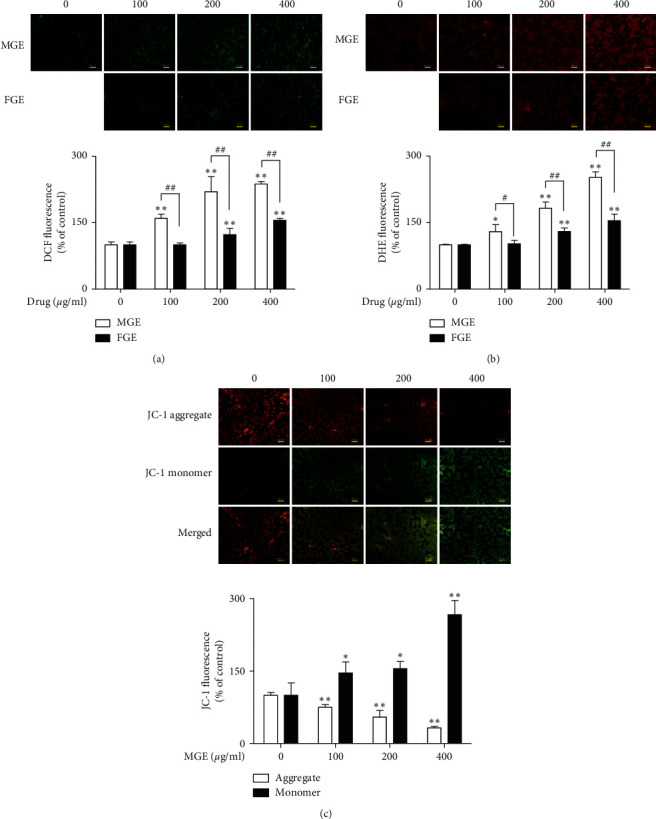
Activatory effects of MGE and FGE on ROS generation and mitochondrial dysfunction in MCF-7 cells. MCF-7 cells were seeded on a black 96-well plate and then further incubated. After 24 h, the cells were preincubated with MGE or FGM for 4 h and then stained with 10 *μ*M DHE, DCFDA, or JC-1 in Hank's balanced salt solution for 30 min in the darkness. ROS generation and mitochondrial dysfunction were determined as described in the Materials and Methods section. Data are expressed as the mean ± SD values of quadruple determinations. ^*∗*^*P* < 0.05 and ^*∗∗*^*P* < 0.01 versus the control group. ^#^*P* < 0.05 and ^##^*P* < 0.01 versus the same concentration group. (a) DCFDA; (b) DHE; (c) JC-1.

To compare the effects of MGE and FGE on the cell viability of human breast cancer cells, we investigated the effects of MGE and FGE on the cell viability of MCF-7 cells. When MCF-7 cells were incubated with MGE or FGE (0–400 *μ*g/ml) for 24 h, both MGE and FGE induced a change in the morphological aspects of MCF-7 cells (Figure 1(a)). In addition, they both dose-dependently inhibited the cell viability of MCF-7 cells (Figure 1(b)). The inhibitory effect of MGE was more potent than that of FGE (Figure 1(b)). Generally, it has been known that the elevation of ROS generation in cancer cells is associated with apoptosis through mitochondrial dysfunction [15]. Thus, we further examined the effects of MGE and FGE on ROS generation and mitochondrial dysfunction in MCF-7 cells. MGE and FGE significantly improved ROS generation in MCF-7 cells (Figures 2(a) and 2(b)). Consistent with the above cytotoxic results, MGE induced more potent ROS formation than FGE in the cells (Figures 2(a) and 2(b)). In addition, MGE dose-dependently increased the fluorescence intensity of JC-1 monomer, a depolarization marker of mitochondria membrane potential [16], while decreasing that of JC-1 dimer, a polarization marker of mitochondria membrane potential [16], in the above cells (Figure 2(c)). These results suggest that MGE has anticancer activity through upregulating ROS generation with mitochondrial dysfunction in human breast cancer. The anticancer effect of MGE was stronger than that of FGE.

### 3.2. Regulatory Effects of MGE on Expression of Apoptosis-Related Proteins in MCF-7 Cells

**Figure 3:**
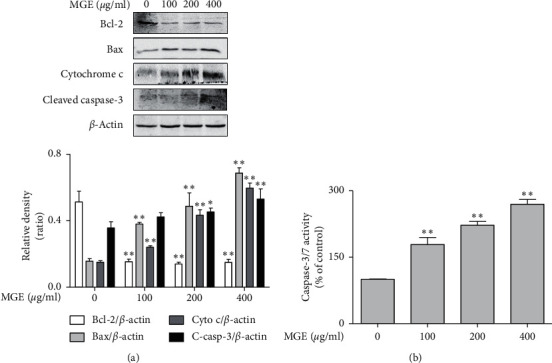
Effect of MGE on expression of apoptosis-related proteins. MCF-7 cells were seeded on a 6-well plate (5 × 10^4^ cells/well) and then further incubated. Next day, the cells were incubated with MGE for 24 h. The expression of Bcl-2, Bax, cytochrome c cleaved caspase-3, or *β*-actin and caspase-3/7 activity was determined as described in the Materials and Methods section. Similar results for immunoblot analysis were obtained in three independent experiments. Data of caspase-3/7 activity are expressed as the mean ± SD values of quadruple determinations.^*∗*^*P* < 0.05 and ^*∗∗*^*P* < 0.01 versus the control group. (a) Bcl-2, Bax, cytochrome c, and cleaved caspase-3; (b) caspase-3/7 activity.

After finding that MGE possessed more potent cytotoxicity than FGE through upregulating ROS generation with mitochondrial dysfunction in human breast cancer, we were concerned that the anticancer action of MGE was associated with the regulation of ROS-mediated apoptosis because elevated ROS generation in cancer cells leads to activation of the caspase-dependent apoptosis pathway [17]. Thus, we further investigated the effect of MGE on ROS-mediated apoptosis in MCF-7 cells. MGE increased the expression of proapoptotic factors, such as Bax, cytochrome c, and cleaved caspase-3, while decreasing the expression of Bcl-2, an antiapoptotic factor, in MCF-7 cells (Figure 3(a)). Furthermore, MGE dose-dependently enhanced the activity of capase-3/7 in the cells (Figure 3(b)). These findings indicate that MGE promotes apoptosis through inducing the generation of excessive oxidative stress in human breast cancer. In addition, the apoptotic effect of MGE is closely correlated with the regulation of the expression of apoptosis-related proteins.

### 3.3. Inhibitory Effect of MGE on the Growth of Human Breast Cancer in Mice

**Figure 4:**
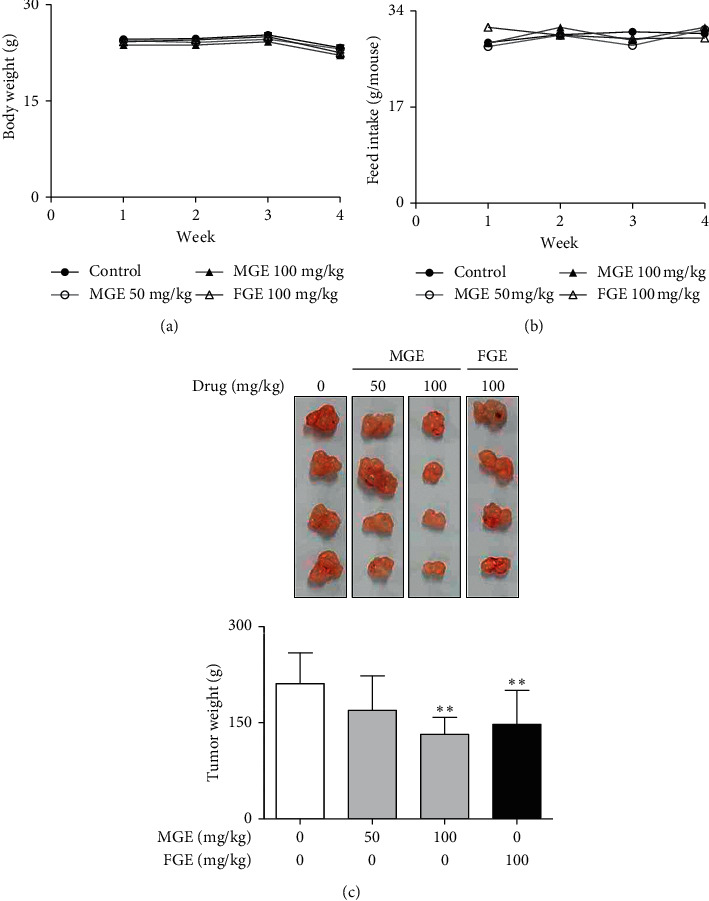
Inhibitory effects of MGE and FGE on the growth of human breast cancer in nude mice. Mice were intravenously administered with MGE or FGE once a day for 4 weeks. Body weights, feed intake, and tumor weights were determined as described in the Materials and Methods section. Data are expressed as the mean ± SEM values of quintuple determinations. ^*∗*^*P* < 0.05 and ^*∗∗*^*P* < 0.01 versus the control group. (a) Body weight; (b) feed intake; (c) tumor weight.

**Figure 5:**
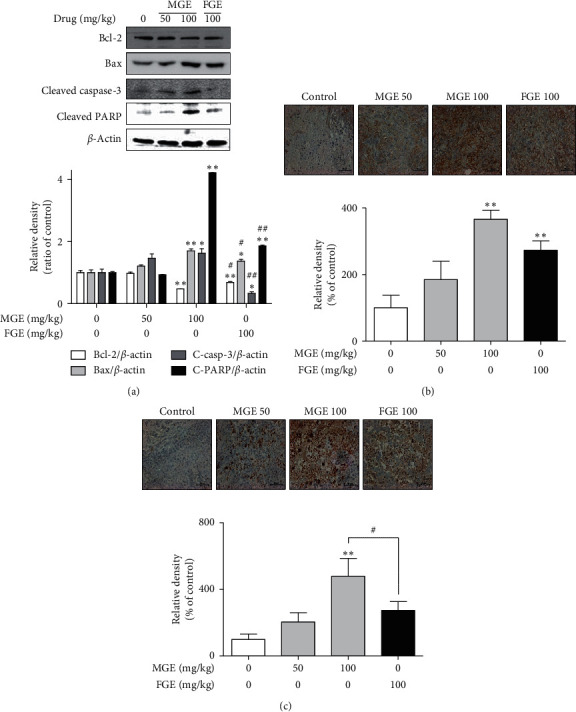
Effects of MGE on expression of apoptosis-related proteins in tumor tissues. Tumor tissues of human breast cancer, isolated from sacrificed mice, were homogenized and then lysed, or the tissues were fixed and then sectioned. Immunoblot analysis and histological analysis were performed as described in Materials and Methods section. Similar results were obtained in three independent experiments. ^*∗*^*P* < 0.05 and ^*∗∗*^*P* < 0.01 versus the control group. ^#^*P* < 0.05 and ^##^*P* < 0.01 versus MGE-treated group (100 mg/kg). (a) Bcl-2, Bax, cleaved caspase-3, and cleaved PARP; (b) Bax; (c) cleaved PARP.

To confirm the apoptotic effect of MGE on human breast cancer in a whole body system, we further examined whether MGE could attenuate the growth of human breast cancer in a xenograft model. When mice were intravenously administrated with MGE or FGE once a day for 4 weeks, MGE did not affect their body weights or food intake (Figures 4(a) and 4(b)). As expected, MGE dose-dependently reduced the increments of tumor weights in the mice (Figure 4(c)). In addition, the inhibitory effect of MGE was more potent than that of FGE. Furthermore, MGE enhanced the expression of Bax, cleaved caspase-3, and cleaved PARP, proapoptotic factors, while reducing the expression of Bcl-2, an antiapoptotic factor, in tumor tissues of human breast cancer (Figure 5(a)). In histological analysis, it was found that the expression of proapoptotic factors, such as Bax and cleaved PARP, was mainly concentrated in the tumor tissues (Figures 5(b) and 5(c)). These results suggest that MGE exerts an anticancer effect on the growth of human breast cancer within nontoxic ranges in the whole body system. Such an effect of MGE is closely associated with the regulation of the expression of apoptosis-related proteins in human breast cancer. Therefore, MGE may have the possibility of being a drug candidate or adjuvant for the treatment of human breast cancer.

### 3.4. Phytochemical Profile of the Active Components in MGE: Comparison between MGE and FGE

To substantiate what is responsible for the anticancer actions of MGE, we analyzed the ginsenoside composition in MGE and FGE using an HPLC system. Then, we compared the ginsenoside levels in MGE and FGE because some ginsenosides derived from farm-cultivated ginseng are known to possess anticancer activities in various cancers [18]. Based on a previous report, we analyzed the ginsenoside contents in MGE and FGE and quantified the amounts of the ginsenosides. The amounts of ginsenosides in MGE and FGE are summarized in Table 1. The ginsenoside levels in MGE were high in the order of Rb1 > Rb2 > Rc > Re > Rg1 > Rg2 > Rf > Rg3 and those in FGE were high in the order of Rb1 > Rg1 > Rb2 > Re > Rc > Rg2 > Rf ≧ Rg3. Additionally, in a comparison of the amounts of each ginsenoside in MGE and FGE, the levels of all the ginsenosides were shown to be higher in MGE than in FGE. Ginsenoside Rf in MGE especially showed a level more than 4 times that in FGE. These findings indicate that MGE is richer in ginsenosides than FGE. In addition, Rb1, Rb2, Rc, Re, and Rg1 may be used as quality index components of mountain ginseng because their amounts are over 1% in MGE and significantly higher than in FGE. Overall, the ginsenosides may be responsible for the anticancer effect of MGE.

## 4. Discussion

Korean ginseng possesses various beneficial effects [2–4] and has been commonly used as an element of traditional medicine and in functional foods around the world. It has been recognized for centuries in Northeast Asia that the beneficial effects of mountain ginseng are better than those of farm-cultivated ginseng. It was reported that mountain ginseng is richer in bioactive phytochemicals than farm-cultivated ginseng [5]. It is presumed that rich bioactive phytochemicals in mountain ginseng are associated with its slow growth by the different cultural environment compared with farm-cultivated ginseng. In fact, mountain ginseng grows up without artificial manipulation after sowing it, whereas farm-cultivated ginseng is cultivated by artificial management after sowing it [5]. Therefore, the cultivated land for mountain ginseng is very limited [19], and the amounts of bioactive phytochemicals in mountain ginseng are variable with the cultural environments of the plant [20]. Although it is still unclarified what is an important key element among environmental factors, influencing the synthesis of phytochemicals in mountain ginseng, currently MGE is being used as a component of pharmacopuncture as a therapy for cancer patients in Korea [10]. However, no evidence has been reported in preclinical studies showing that MGE exerts an anticancer activity.

In the present study, we investigated whether MGE was able to suppress the growth of human breast cancer in *in vitro* and *in vivo* studies, and we found stronger anticancer activity with MGE than with FGE in human breast cancer cells. In addition, MGE led to apoptosis of human breast cancer cells through upregulating ROS generation in the cells. Along with the *in vitro* results, MGE dose-dependently increased the reduction of tumor mass of human breast cancer through regulating expression of apoptosis-related proteins in a xenograft model. Furthermore, the anticancer action of MGE was more potent than that of FGE, while the content of all ginsenosides was higher in MGE than in FGE. Therefore, the anticancer effect of MGE may be closely associated with its richness in ginsenosides because some ginsenosides have anticancer activities [18].

One possible mechanism for the anticancer properties of MGE may be associated with the initiation of apoptosis by promotion of intracellular ROS generation in human breast cancer cells. Generally, the elevation of intracellular ROS with mitochondrial dysfunction leads to induce apoptosis [21]. Anticancer drugs that are used in clinics are able to activate the formation of intracellular ROS with mitochondrial dysfunction in cancer cells [17]. The elevated intracellular ROS activates the expression of Bax, a proapoptotic factor, and also inhibits the expression of Bcl-2, an antiapoptotic factor, in mitochondria [22]. Consequently, the mitochondria liberate cytochrome c into the cytosol, and then the released cytochrome c initiates caspase-dependent apoptosis through activation of the caspase-3/PARP pathway [23]. Therefore, upregulating the intracellular ROS formation in human cancer cells may be an important point for cancer therapy. In support of this, MGE not only promoted the formation of intracellular ROS with mitochondrial dysfunction, but also activated apoptosis through regulating the expression of Bcl-2, Bax, cytochrome c, and cleaved caspase-3 in human breast cancer cells. In addition, MGE reduced the tumor mass of human breast cancer by regulating the expression of apoptosis-related proteins, such as Bcl-2, Bax, cleaved caspase-3, and cleaved PARP, in a whole body system. Overall, the results indicate that MGE has anticancer activity through activating the caspase-dependent apoptosis signaling cascade by the elevation of intracellular ROS generation with mitochondrial dysfunction in human breast cancer.

Another possible mechanism correlated with the anticancer effect of MGE may derive from its richness in ginsenosides. This is because some ginsenosides, such as ginsenosides Rb1, Re, Rg2, Rg3, and Rg5, are known to possess anticancer activity through regulating proliferation and apoptosis in a variety of cancers [18]. In support of this, the anticancer effect of MGE was stronger than that of FGE in both *in vitro* and *in vivo* models. In addition, most of the ginsenosides, except Rg2, Rf, and Rg3, recorded over 10 mg/g dry weight in MGE. Moreover, the contents of ginsenosides Rb1, Rb2, Rc, Re, Rg2, and Rf in MGE were over 2-fold higher than those in FGE. Ginsenoside Rg2 especially, which exerts an anticancer action [18], was over 3-fold higher in MGE than in FGE. This indicates that the anticancer properties of MGE are closely associated with the richness of all the ginsenosides in MGE and may be related to synergistic effects of the ginsenosides. In addition, the ginsenoside profiles in MGE may be useful for evaluating the quality of mountain ginseng.

## 5. Conclusions

This study demonstrates that MGE exerts stronger anticancer properties than FGE in human breast cancer cells in both *in vitro* and *in vivo* models. The anticancer mechanism of MGE is closely associated with boosting the apoptosis signaling cascade by the upregulation of ROS generation in the cells. Therefore, intracellular targets for the anticancer action of MGE include Bcl-2, Bax, cytochrome c, caspase-3, and PARP in human breast cancer. Such anticancer effects of MGE may be associated with its richness in ginsenosides and provide novel information for understanding how MGE suppresses the growth of human breast cancer. Furthermore, this study may support the possibility of using MGE as a herbal drug for therapy of breast cancer patients in clinics. However, other preclinical studies using various human cancer cell lines are necessary to completely support any clinical application because this study has some limits in representing a preclinical study for clinical application. Further, other studies, such as metabolism, pharmacokinetics, toxicology, and phytochemical studies, are necessary to ensure patient safety against MGE and to identify the active compounds in MGE having an anticancer effect.

## Figures and Tables

**Table 1:** Composition of ginsenosides in MGE and FGE.

	Dry weight (mg/g)		
	MGE	FGE	Ratio (MGE/FGE)
Rb1	27.03 ± 0.26	11.70 ± 1.05	2.31 ± 0.02
Rb2	19.76 ± 0.25	7.20 ± 0.31	2.74 ± 0.03
Rc	15.18 ± 0.12	4.91 ± 0.07	3.09 ± 0.03
Re	14.91 ± 0.28	6.00 ± 0.24	2.49 ± 0.05
Rg1	11.17 ± 0.06	7.52 ± 0.08	1.49 ± 0.01
Rg2	2.81 ± 0.04	0.86 ± 0.01	3.26 ± 0.05
Rf	1.88 ± 0.04	0.43 ± 0.01	4.36 ± 0.09
Rg3	0.32 ± 0.02	0.28 ± 0.00	1.13 ± 0.06

Data are expressed as mean ± SD values of triple determinations.

## Data Availability

All data used to support the findings of this study are included in the article.

## Abbreviations

Bax: Bcl-2-associated X protein
Bcl-2: B-cell lymphoma 2
FGE: Farm-cultivated ginseng extract
MGE: Mountain ginseng extract
PARP: Poly (ADP-ribose) polymerase
ROS: Reactive oxygen species.

## Supplementary Materials

Supplementary Materials Supplementary Figure 1: HPLC chromatograms of MGE and FGE. Ginsenosides Rg1 (1), Re (2), Rf (3), Rb1 (4), Rc (5), Rb2 (6), Rg2 (7), and Rg3 (8) were identified. HPLC analysis was performed as described in Materials and Methods section. A, MGE chromatogram; B, FGE chromatogram.
