# Supplementary material for: Anticancer Effect of Mountain Ginseng on Human Breast Cancer: Comparison with Farm-Cultivated Ginseng
Source: Evid Based Complement Alternat Med. 2020 Jul 25;2020:2584783. doi: 10.1155/2020/2584783 (PMC7399781; doi:10.1155/2020/2584783)
Supplement: Supplementary Materials — Supplementary Figure 1: HPLC chromatograms of MGE and FGE. Ginsenosides Rg1 (1), Re (2), Rf (3), Rb1 (4), Rc (5), Rb2 (6), Rg2 (7), and Rg3 (8) were identified. HPLC analysis was performed as described in Materials and Methods section. A, MGE chromatogram; B, FGE chromatogram. [file 2584783.f1.pdf]

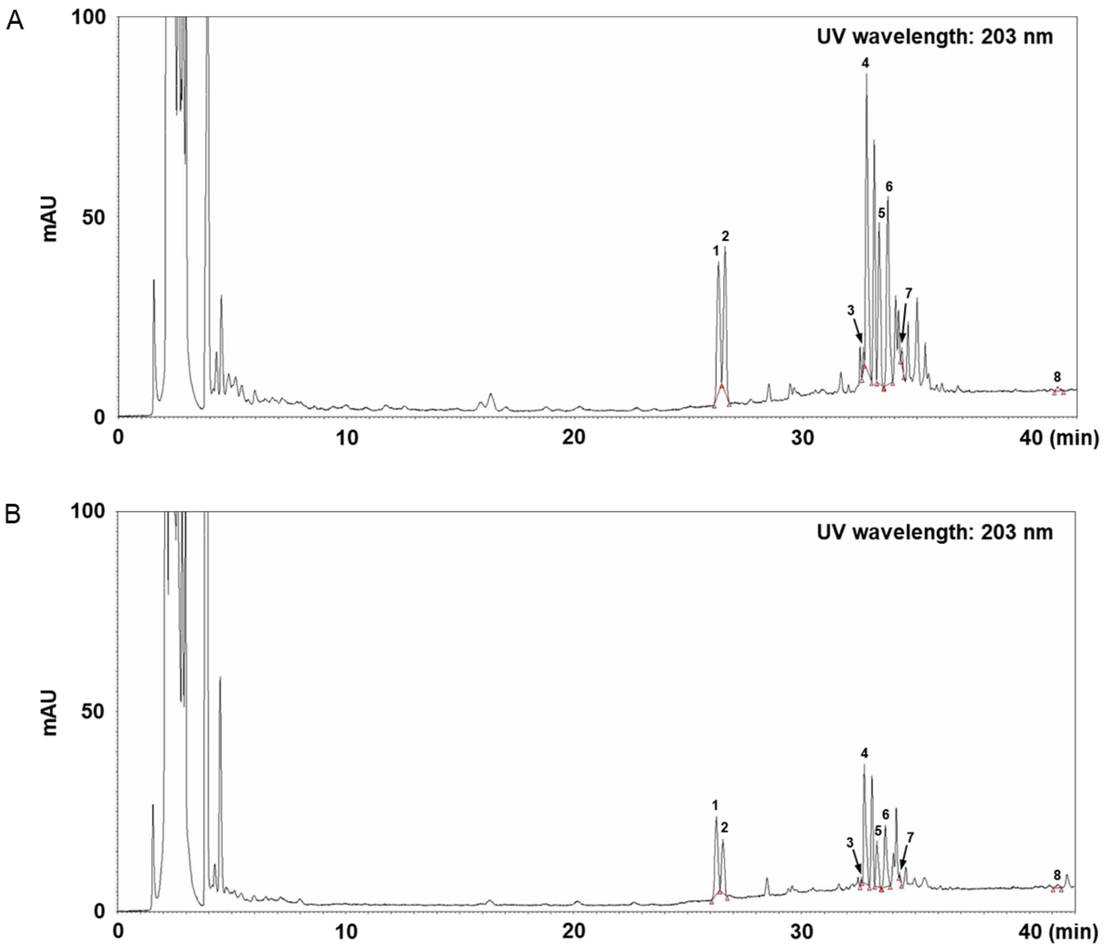

Supplementary Figure 1. HPLC chromatograms of MGE and FGE.

Ginsenoside Rg1 (1), Re (2), Rf (3), Rb1 (4), Rc (5), Rb2 (6), Rg2 (7) and Rg3 (8) were identified. HPLC analysis was performed as described in the Materials and methods section. A, MGE chromatogram; B, FGE chromatogram.
